# 309. Detection of hemagglutinin H5 influenza A virus sequence in municipal wastewater solids during an outbreak of avian influenza

**DOI:** 10.1093/ofid/ofae631.099

**Published:** 2025-01-29

**Authors:** Alessandro Zulli, Marlene Wolfe, Alexandria Boehm

**Affiliations:** Stanford University, Stanford, CA; Emory University; Stanford University, Stanford, CA

## Abstract

**Background:**

Influenza is a significant cause of illness and death worldwide. Influenza A virus is responsible for all influenza epidemics, and some strains, such as highly pathogenic avian influenza (HPAI), have zoonotic reservoirs and potential. In 2024, an outbreak of HPAI H5N1 clade 2.3.4.4b, spread to dairy cattle in the United States, raising concerns about potential contributions to wastewater from animal sources. Wastewater surveillance has become a routine tool for monitoring influenza outbreaks due to providing population-level data in near real-time, making it uniquely suited to detection of emerging pathogens.

H5 gene concentrations in wastewater solids during cattle outbreaks of avian influenza, Texas

**Figure 1.**
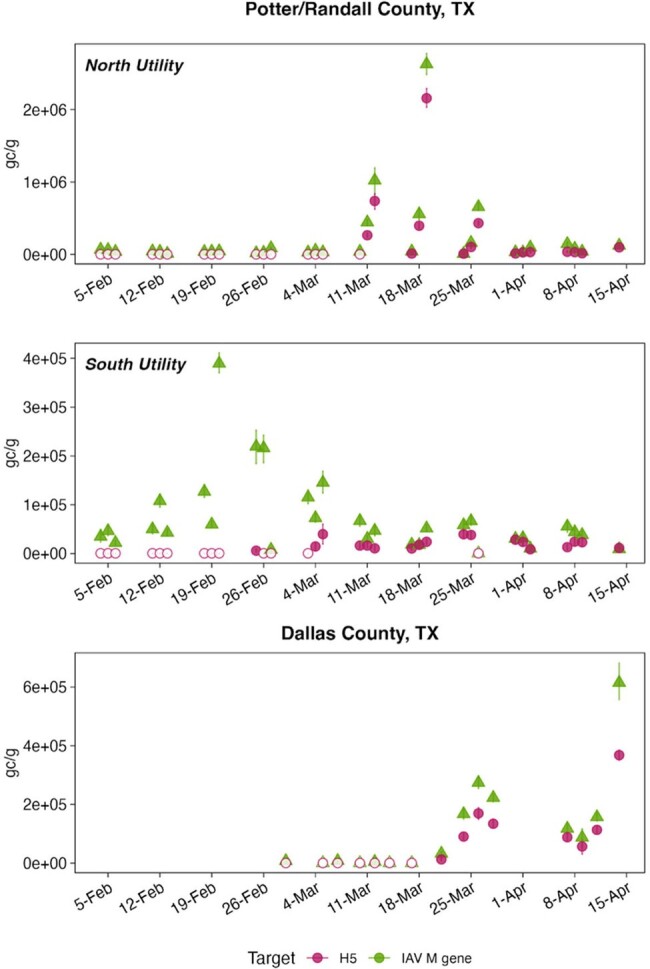
Concentrations of IAV M gene target (labeled as IAV, in green) as well as the H5 marker (labeled H5) and associated standard error in units of copies per gram dry weight; if error bars cannot be seen, then they are smaller than the symbols. Open markers are below the lowest measurable concentration (1000 copies per gram dry weight).

**Methods:**

We developed a hydrolysis probe digital droplet RT-PCR assay for the detection of the H5 hemagglutinin gene of IAV and demonstrated it to be 100% specific and 90% sensitive based on in-silico and in-vitro testing against panels of human viruses including other IAV subtypes. We identified wastewater treatment plants (WWTPs) with aseasonal increases in influenza A concentrations. Clinical data was aggregated from influenza-related emergency health visits and statewide positivity rates and used for comparison to measured influenza concentrations.

A timeline of events related an avian influenza outbreak in dairy cattle

**Figure 2.**
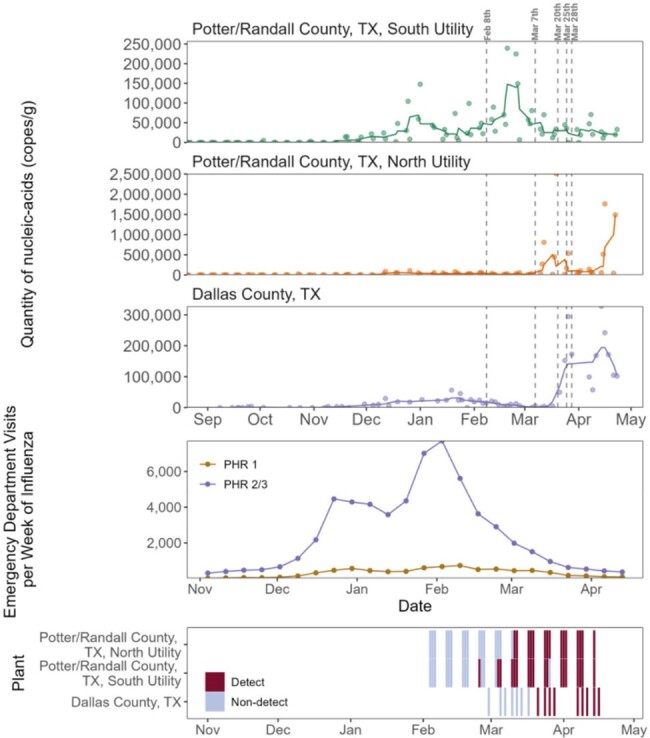
Top three panels. Concentrations of the IAV M gene in wastewater solids at the three WWTPs in Texas, as measured in the prospective monitoring. Line represents the 5-adjacent sample trimmed average, the symbols show the measured concentrations and the standard errors. Fourth panel from the top shows emergency department visits per week for influenza; PHR 1 represents Texas Public Health Region 1 (containing Potter/Randall Counties) and PHR2/3 represents Public Health Region 2/3 containing Dallas County. Bottom panel shows the presence or absence of the H5 marker in the samples from the three plants. Vertical lines indicate key dates as follows. February 8, 2024 USDA declares ongoing HPAI poultry outbreak, March 7, 2024: Unknown dairy cattle illness first reported, March 20, 2024: Samples collected from dairy cattle in Texas, March 25, 2024: Texas confirms H5N1 in dairy cattle, March 28, 2024: Positive H5N1 specimen collected from human.

**Results:**

During the monitoring period, 59 WWTPs showed increases in the IAV M gene after the seasonal influenza period that coincided with the emergence of HPAI H5N1 in dairy cattle. Retrospective testing of samples from three WWTPs in Texas revealed the presence of the H5 marker at concentrations approaching those of the IAV M gene. The detection of the H5 marker in wastewater occurred before the official confirmation of H5N1 in dairy cattle and detection of a human case in Texas. Two of the WWTPs in Texas were confirmed to have permitted discharges from animal product processing facilities, including dairies, which is the proposed source of the H5 signal in wastewater.

**Conclusion:**

This study demonstrates the ability of wastewater monitoring to detect contributions from zoonotic influenza, highlighting the need to consider industrial and agricultural inputs into wastewater systems. The detection of H5 in wastewater could have provided an early warning of the HPAI H5N1 outbreak before official confirmation, illustrating the value of wastewater surveillance when dealing with rapidly emerging pathogens.

**Disclosures:**

**All Authors**: No reported disclosures

